# ITIH4: A New Potential Biomarker of “Toxin Syndrome” in Coronary Heart Disease Patient Identified with Proteomic Method

**DOI:** 10.1155/2013/360149

**Published:** 2013-08-19

**Authors:** Hao Xu, Qinghua Shang, Hao Chen, Jianpeng Du, Jianyan Wen, Geng Li, Dazhuo Shi, Keji Chen

**Affiliations:** ^1^Xiyuan Hospital, China Academy of Chinese Medical Sciences, Beijing 100091, China; ^2^Beijing University of Chinese Medicine, Beijing 100029, China; ^3^Wuxi Hospital of Traditional Chinese Medicine, Wuxi 214001, China; ^4^China-Japan Friendship Hospital, Beijing 100029, China

## Abstract

*Objective*. This trial aims to look for the protein biomarker of “toxin syndrome” of CHD patients. *Methods*. We have performed two trials in this paper. The first trial was a randomized controlled trial (RCT) of the plasma proteome in unstable angina (UA) patients by Maldi-Tof Mass. The second trial was a nested case-control study in 1503 stable CHD patients with one-year followup for acute cardiovascular events (ACEs). *Results*. In the RCT study, 12 protein spots were found to be the differential protein for the significant differences between the difference of before and after treatment in group A and group B; 2 of them (3207.37 Da and 4279.95 Da) was considered to be unique to “toxin syndrome” for being differential proteins of group B but not group A. These 2 spots were identified as Isoform 1 of Fibrinogen alpha chain precursor (FGA, 3207.37 Da) and Isoform 2 of inter-alpha-trypsin inhibitor heavy chain H4 (ITIH4, 4279.95 Da), respectively. In the nested case-control study, the result of Western blot demonstrated that protein expression of ITIH4 in the group with followup ACEs was significantly lower than the matched group without followup ACEs (*P* = 0.027). *Conclusion*. ITIH4 might be a new potential biomarker of CHD “toxin syndrome” in TCM, indicating the potential role in early identifying high-risk CHD patients in stable period.

## 1. Introduction

Syndrome differentiation is a unique diagnostic method of traditional Chinese medicine (TCM) [1, 2]. “Blood stasis syndrome” (BSS) is considered as a major and key syndrome in the process of coronary heart disease (CHD) in TCM [3, 4], and activating blood circulation and dissolving stasis has been a mainstream treatment for CHD. However, some stable CHD patients develop acute cardiovascular events (ACEs), while others do not, why? Based on this question, we proposed a hypothesis of “blood stasis and toxin” considering blood stasis was a constant pathogenesis in CHD, while “toxin” was the trigger in transforming to ACEs [5].

The original meaning of “toxin” is a kind of poisonous herb but it has been considered as a pathogenic factor in a narrow sense and pathogenesis, medicine, and syndrome in a broad sense. It is often seen in the fields of epidemic febrile diseases and surgical diseases (such as carbuncle, abscess, hard furuncle, and sore). Zhang et al. [6] presented a theory of “artery carbuncle” according to previous studies that arteriosclerosis plaque has the characteristics such as redness, swelling, and being hot on the local scale, just like the traditional “toxin syndrome.” Heat-clearing and detoxifying treatment has been widely used in CHD, especially acute coronary syndrome patients [7–9]. Previous studies showed that Rhizoma Coptidis, Cyrtomium Rhizome, compound simiaoyongan decoction, and Huanglian Jiedu decoction could improve clinical symptoms by multiple mechanisms such as anti-inflammatory action, lipid regulation, and AS plaque reduction [10–19]. Furthermore, a lot of researches indicated that drugs for activating blood circulation and detoxifying had a better effect on relieving angina than drugs for activating blood circulation only; it might be related to the effect of anti- inflammatory action [20–27].

Changes in macroscopic manifestation certainly have the corresponding microscopic biological basis. Inflammation has been proved to be a biomarker for CHD/ACS, and the proteome supported us with a new technology for studying it further. The proteome is a subject studying all the proteins in a cell, a kind of tissue, or an organism in specific conditions or at specific times and has been one of the most potential and effective approaches for decoding and revealing the biological foundation and essence of syndromes. Different syndromes consequentially have relevant differential protein expressions; meanwhile, one syndrome also has different protein expressions after treatment of different medicines. Therefore, the protein's characteristics of a specific syndrome can be reflected by the effectiveness of prescriptions corresponding to syndromes. 

Berberine extracted from Rhizoma Coptidis, a representative herb of clearing heat and detoxifying, could inhibit the expressions of inflammatory factors such as thromboxane A2 and prostaglandin I2 after the injury of blood vessels [10]. Xiongshao capsule, consisting of active ingredients ( Chuanxiongol and paeoniflorin), has shown beneficial effect in atherosclerosis or CHD in clinical and experimental studies [28–34]. Therefore, it was served as a representative Chinese medicine for activating blood circulation.

The aim of this study was to look for the protein biomarker of “toxin syndrome” of CHD patients, which is anticipated to help early identification of high-risk CHD patients in stable period.

## 2. Design and Ethics Statement

**Figure 1:**
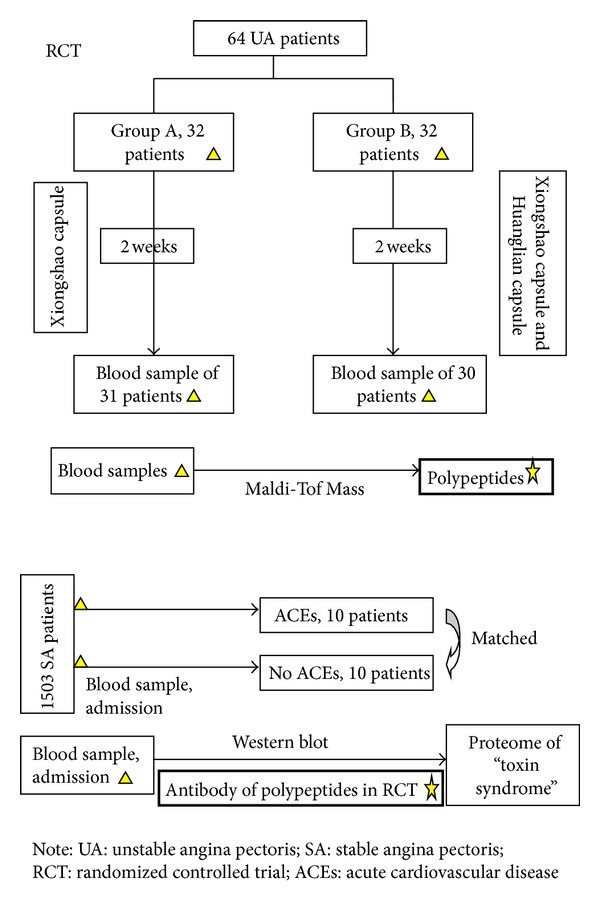
Flow chart of the study.

There are two parts in this paper (Figure 1). The first one was a randomized controlled trial (RCT) with 2 study groups conducted at 2 cooperating hospitals (Anzhen Hospital and Tongren Hospital) to look for biomarkers for “toxin syndrome” of TCM. The other was a nested case-control study with a follow-up for ACEs conducted at 5 cooperating hospitals (China Academy of Chinese Medical Sciences Xiyuan Hospital, China-Japan friendship Hospital, Anzhen Hospital, Tongren Hospital, and Fujian Integrative Medicine Clinic) to verify the biomarker found in RCT. The trials were carried out according to the Declaration of Helsinki, and the protocols were approved by the institutional review boards and ethics committees at each center. All the patients provided written informed consent.

## 3. Materials and Methods

### 3.1. Randomized Controlled Trial

#### 3.1.1. Patients

Fasting serum samples were obtained from 64 patients with UA (ICD-10  :  I20.0/20.1/20.9) [35, 36] aged between 40 and 75 years old. All of the patients who were admitted into the 2 cooperating hospitals were enrolled in the study. The inclusion criteria were successful PCI in 48 hours after the first severe angina and BSS of TCM (including Qi-stagnation-blood-stasis syndrome and Qi-deficiency-blood-stasis syndrome) [37–39]. Patients were excluded if they met any of the following criteria: presence of (1) stable angina or acute myocardial infarction; (2) inflammation, fever, trauma, bure, or surgery in one recent month; (3) active tuberculosis or rheumatic autoimmune disease; (4) known renal insufficiency and serum creatinine >2.5 mg/dL in male and >2.0 mg/dL in female; (5) known hepatic insufficiency and alanine transaminase (ALT) > three times value of the normal level; (6) severe heart failure (EF < 35%); (7) complication by severe primary disease such as hematologic systems and psychological abnormalities; (8) malignancies; (9) organ transplantation; (10) participants of other clinical trials; (11) taking other Chinese patent drugs; (12) pregnancy or breast-feeding. Patients were removed from analysis if they could not estimate the efficacy for they did not take any medicine or did not participate reexamination as proposal.

#### 3.1.2. Groups and Drugs

64 eligible patients were randomly assigned in a 1:1 ratio to group of activating blood circulation (Xiongshao capsule, Z20053499, Hospital preparation approved by Beijing drug administration, group A) or group of activating blood circulation and detoxification (Huanglian capsule, Z19983042, Hubei Xianglian Pharmaceutical Co.,Ltd and Xiongshao capsule, group B). Randomization table was performed centrally with the use of SAS software and reserved by a specific person who did not participate this clinic research. Randomized number was obtained by telephone if any patient was eligible.

In the group of activating blood circulation, Xiongshao capsule was taken as 500 mg (2 capsules) 3 times per day for 2 weeks. In the group of activating blood circulation and detoxification, Huanglian capsule was taken as 500 mg (2 capsules) 3 times per day, and Xiongshao capsule was taken as 500 mg (2 capsules) 3 times per day for 2 weeks.

All patients received western standardized medication including antiplatelet drugs (aspirin and/or clopidogrel hydrogen sulfate), anticoagulant drugs (heparin or low molecular weight heparin), anti-ischemic drugs (nitrates, *β*-blocker, calcium channel blocker, and angiotensin-converting enzyme inhibitors), and statins. 

#### 3.1.3. Data Collection

At the beginning of the trial, all patients filled out a standardized questionnaire containing general information, past history, risk stratification of UA [40], angina score, primary symptom score of TCM [41], BSS score [42], medical treatment, and PCI surgery. In addition, to obtain the serum, at the beginning and the end of the trial, 2 ml of blood from each patient with an empty stomach was drawn into common coagulation-promoting tubes, centrifuged at 3000 r for 10 min at room temperature to remove insoluble materials, cells, and debris, and supernatants were kept at −80°C until use. 

#### 3.1.4. Reagents and Instruments

The WCX magnetic bead kit (Bruker Daltonics Tech, Beijing, China), alpha-cyano-4-hydroxycinnamic acid (HCCA), MALDI-TOF MS (type: microflex, Bruker Daltonics Biosciences, Bremen, Germany), 100% ethanol (chromatographic grade), and 100% acetone (chromatographic grade) were freshly prepared (sigma).

#### 3.1.5. WCX Fractionation and MALDI-TOF MS Analysis

The suspension in the WCX magnetic bead kit was mixed by shaking. After eluting and beating, the magnetic beads were separated from the protein, and the eluted peptide samples were transferred to a 0.5 mL clean sample tube for further MS analysis. Five microliters of HCCA substrate solution (0.4 g/L, dissolved in acetone and ethanol) and 0.8–1.2 *µ*L of elution were mixed. Then, 0.8–1.2 *µ*L of this mixture was applied to a metal target plate and dried at room temperature. Finally, the prepared sample was analyzed by MALDI-TOF MS. A range of 1000–10,000 Da peptide molecular weights was collected, and 400 shots of laser energy were used. Peptide mass fingerprints were obtained by accumulating 50 single MS signal scans.

#### 3.1.6. Peptide Sequence

Experiment for 4280.13 *m/z* peptide identification was performed using a nano-liquid chromatography-electrospray ionization-tandem mass spectrometry (nano-LC/ESI-mass spectrometry/mass spectrometry) system consisting of an Acquity UPLC system (Waters) and an LTQ Orbitrap XL mass spectrometer (Thermo Fisher) equipped with a nano-ESI source. The peptide solutions were loaded to a C18 trap column (nano-Acquity) (180 *μ*m × 20 mm × 5 *μ*m (symmetry)). The flow rate was 15 *μ*L/min. Then the desalted peptides were analyzed by C18 analytical column (nano-Acquity) (75 *μ*m × 150 mm × 3.5 *μ*m (symmetry)) at a flow rate of 400 nL/min. The mobile phases A (5% acetonitrile, 0.1% formic acid) and B (95% acetonitrile, 0.1% formic acid) were used for analytical columns. The gradient elution profile was as follows: 5%B–50%B–80%B-80%B–50%B–5%B in 100 min. The MS instrument was operated in a data-dependent model. The range of full scan was 400–2000 *m/z* with a mass resolution of 100,000 (*m/z* 400). The eight most intense monoisotope ions were the precursors for collision induced dissociation. Mass spectrometry was limited to two consecutive scans per precursor ion followed by 60 s of dynamic exclusion.

#### 3.1.7. Statistical Analysis

ClinProTools (ClinProt software version 2.1, Bruker Daltonics) was used to subtract baseline, normalize spectra (using total ion current), and determine peak *m/z* values and intensities in the mass range of 1000 to 10,000 Da. The signal-to-noise (S/N) ratio should be higher than five. To align the spectra, a mass shift of no more than 0.1% was determined. The peak area was used as quantitative standardization. Student's *t*-test was used for analysis of normally distributed continuous data, while Wilcoxon test for nonnormally distributed continuous data. Chi-square test was used for categorical data analysis. A *P* value <0.05 was considered significant. 

### 3.2. Nested Case-Control Study

#### 3.2.1. Patients

1503 patients with stable CHD (old myocardial infarction or at least one significant (>50%) stenosis that was documented on a recent coronary angiogram and WHO [35]) younger than 80 years old were enrolled from 5 cooperating hospitals. Stable CHD was defined as no symptoms or stable exertional angina or patients in stable condition after ACS for at least 1 month. Patients were excluded if they met any of the following criteria: presence of (1) inflammation, fever, trauma, bure, or surgery in one recent month; (2) active tuberculosis or rheumatic autoimmune disease; (3) severe heart failure (EF < 35%); (4) complication by severe valvular heart disease, or myocardiopathy; (5) complication by severe chronic obstructive pulmonary disease (COPD), pulmonary heart disease or respiratory failure; (6) known renal insufficiency and serum creatinine >2.5 mg/dL in male and >2.0 mg/dL in female; (7) known hepatic insufficiency and alanine transaminase (ALT) > three times value of the normal level; (8) complication by severe primary disease such as hematologic systems; (9) severe psychological abnormalities; (10) malignancies; (11) viscera transplantation; (11) life expectancy less than 3 years. Patients were removed from analysis if a mistaken inclusion or lack of necessary record for analysis or failure to follow up for ACEs because of missing contact information took place.

#### 3.2.2. Data Collection

In all patients, follow-up was scheduled at 0.5 and 1 year after inclusion of the trial. At every visit of the trial, information was obtained from each patient by use of a standardized questionnaire, the information regarding general information, past history, and the secondary cardiovascular events in follow-up. Physicians collecting information were unaware of the purpose of the study. Secondary cardiovascular events were defined as death from heart disease, nonfatal myocardial infarction (MI), or ischemic cerebrovascular events (stroke or transient ischemic attack). All the cardiovascular events were estimated by consulting medical records. In addition, the serum also was collected at every visit, and the method of blood collection, centrifugation, and storage was the same as that of RCT. 

Twenty three patients were confirmed as ACEs during one-year follow-up, and 10 patients were selected for their well preserved serum sample. Another 10 patients with no follow-up ACEs were matched in a 1 : 1 ratio by sex, age (±5 years), hypertension history, diabetes history, and myocardial infarction history. All the sera at the admission of these 20 patients were adopted for verifying the differential protein of “toxin syndrome” obtained from RCT by Western blot method.

#### 3.2.3. Western Blot

To detect the inter-alpha-trypsin inhibitor heavy chain H4 (ITIH4) obtained from RCT (see results section), blood serum stored in −80°C refrigerator was assayed using Western blot as described before [43]. Additionally, ITIH4 antibody (1 : 2500, Sigma, USA) was used for detection of ITIH4. The horseradish peroxidase (HRP) conjugated anti-mouse IgG (0.1 mL/cm^2^, Santa Cruz Biotechnology, UAS) was used as the secondary antibody, and signals were visualized using the enhanced chemiluminescence system (ECL, Pierce, USA).

### 3.3. Statistical Analysis

Statistical analysis was performed by a statistician in a blind fashion. Statistical analysis was performed with SPSS15.0 software. All tests were two tailed, and a statistical probability of <0.05 was considered significant. Normality test and homogeneity test of variances were conducted. Frequency table, percentage or constituent ratio for describing enumeration data; $\overset{-}{X}\pm S\;\;$for describing measurement data. *χ* ^2^ test or Fisher exact test if necessary was used for comparison of enumeration data, *t*-test was used for comparison of measurement data (corrected *t* test was used if variant heterogeneity), and Wilcoxon tests were used for abnormal distribution.

## 4. Results

### 4.1. Patients' Characteristics in RCT

64 participants with UA were enrolled in 5 centers and were randomized into two groups: 32 to receive Xiongshao capsule (group A) and 32 to receive Xiongshao capsule and Huanglian capsule (group B). During the course of the study, one patient was excluded in group A due to incomplete follow-up, while two patients were excluded in group B with 1 incomplete follow-up, and 1 noncompliance with medications. They were removed from statistics as the stated protocol. Thus finally, the population in analysis consisted of 61 patients, with 31 patients in group A and 30 patients in group B. The baseline characteristics of the UA patients were summarized in Table 1. The two groups were well matched with regard to baseline clinical and angiographic characteristics (*P* > 0.05). 

### 4.2. Sample Processing in RCT

During the course of the protein analysis, five blood samples were excluded from group A due to bad peptide mass spectrometry; thus, the population in differential protein analysis consisted of 56 patients, with 26 patients in group A and 30 patients in group B. Acquisition mass range 500–10000 Da (low-to-medium molecular mass range) would be studied in bioinformatics analysis.

### 4.3. MALDI-TOF Mass Spectrometry Analysis of Peptides in Serum of RCT

**Figure 2:**
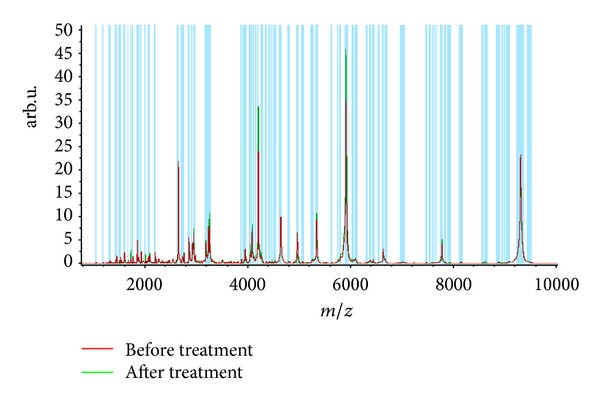
Peptide mass spectrometry before and after treatment in group A.

**Figure 3:**
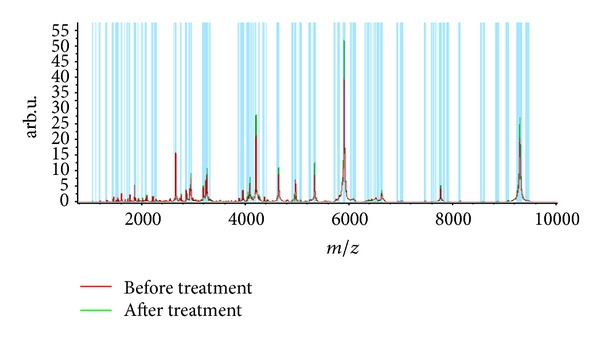
Peptide mass spectrometry before and after treatment in group B.

**Figure 4:**
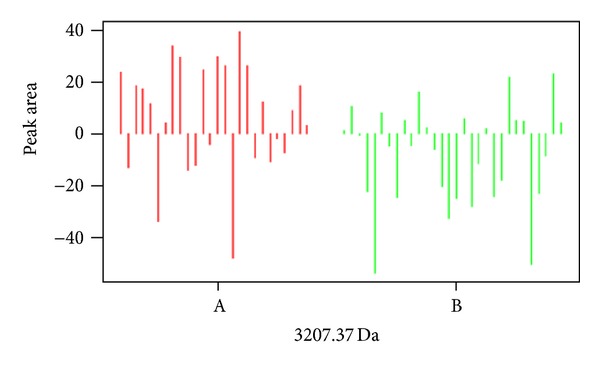
Difference of 3207 Da protein peak between group A and group B.

**Figure 5:**
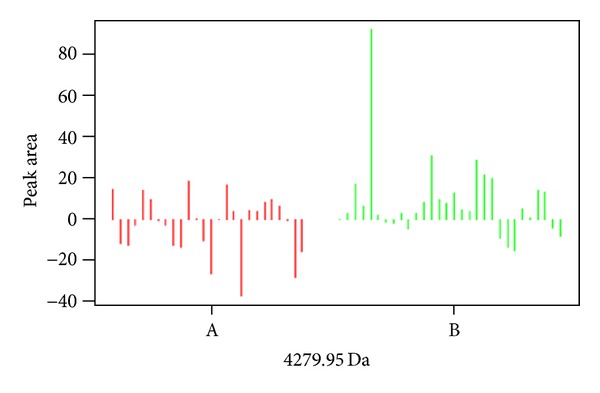
Difference of 4279.95 Da protein peak between group A and group B.

Statistical analysis of the data revealed that the expression of 24 spots was altered after treatment as compared with that at admission in group A (7 of them upregulated and 17 downregulated, Table 2, Figure 2). The expression of 15 spots was altered after treatment as compared with that at admission in group B (8 of them upregulated and 17 downregulated, Table 3, Figure 3), and 4 of the 15 spots were the same as group A. Twelve protein spots were found (Table 4) to be the differential protein for the significant differences between the difference of before-after treatment in group A and group B; 2 of them (3207.37 Da and 4279.95 Da) were considered to be unique to “toxin syndrome” for being differential proteins of group B but not group A. These 2 spots were identified by mass spectrometry (Figures 4 and 5).

### 4.4. Identification of Protein Fragments by Proteome Analysis in RCT

Isoform 2 of inter-alpha-trypsin inhibitor heavy chain H4 (ITIH4) and Isoform 1 of Fibrinogen alpha chain precursor (FGA) were identified in different spots by proteome analysis which can be served as biomarkers of “toxin syndrome” in CHD patients (Table 5).

### 4.5. Western Blot

**Figure 6:**
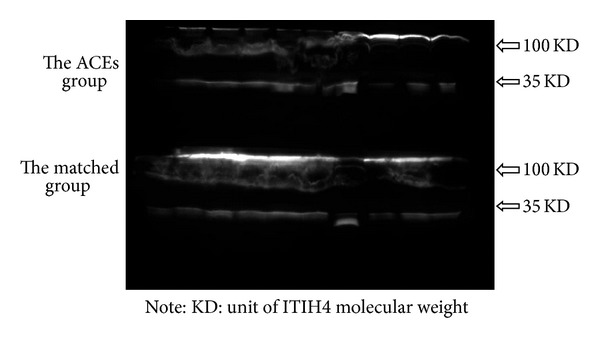
ITIH4 expression between the ACEs group and the Matched group.

A large multicenter nested casecontrol study was conducted for verifying the unique protein biomarker to “toxin syndrome” of TCM obtained from RCT. The admission blood samples of 20 patients were collected for Western blot (10 patients, resp. in the ACEs group and the matched group). We assay the serum protein concentrations and based on the readings load the same amount of protein. In the posttranslational process, the protein ITIH4 was modified and cleaved by plasma kallikrein to yield 100 kDa and 35 kDa fragments. Statistics indicated that protein expression of ITIH4 in the ACEs group was significantly lower than that in the matched group (*P* = 0.027) (Table 6, Figure 6). Therefore, the results of nested case-control study further demonstrated the biomarker identified in the RCT, which indicated that the reduced ITIH4 might be a unique protein biomarker/bioinformation of “toxin syndrome” in CHD patients.

## 5. Discussion

As the development of systems biology and the advancement of the human genome project increased, more and more attention has been paid on the importance of proteome. In this study, we identified 2 peptides (FGA and ITIH4) related to CHD “toxin syndrome” by “taking special drugs to ascertain syndromes” in RCT using MOLDI-TOF MS. Since fibrinogen has been proven to be a risk factor for ACEs in many previous studies [44–46], we only verified another differential protein, ITIH4, by Western blot in a subsequent nested case-control study. Finally, ITIH4 was ascertained to be a new biomarker for CHD “toxin syndrome,” which can also be served as a new risk predictor for ACEs in stable CHD patients.

BSS is one of the basic syndromes in CHD, and “toxin syndrome” is the key in pathogenesis of disease progression. BSS and “toxin syndrome” can coexist or transform to each other, which make up the whole pathological process of CHD [5]. From the macroscopic point of view, Xu et al. [47, 48] enrolled 254 stable CHD patients, collected the clinical information and ACEs in follow-up, and thus concluded a series of clinical manifestations for “toxin syndrome” in stable CHD patients including pain in substernal, headache, uneven or irregular pulse, frequent pharyngalgia, and increased high-sensitivity C-reactive protein (hs-CRP). Other scholars [49] collected clinical information and then summarized a differentiation standard for “toxin-stasis syndrome” of ACS in Chinese medicine. From the microcosmic point of view, “inflammatory reaction” is always a research focus for CHD “toxin syndrome.” Wen et al. [23] investigated the effect on plaque stabilization among herbs regulating blood circulation (Salvia Miltiorrhiza, Radix Paeoniae Rubra), activating blood circulation (Szechuan Lovage Rhizome, Panax Notoginseng), and breaking blood stasis (Peach Seed, Rhubarb Root Parched in Wine) from inflammation, pathomorphology, cellular composition, and so on; indicated Rhubarb root parched in wine was the best for its effect of breaking blood stasis and detoxification. Zhou et al. [26] proposed the hypothesis of “activating blood circulation and detoxification—inflammatory reaction inhibition—plaque stabilization,” then compared the effect on plaque stabilization among herbs activating blood circulation (Panax Notoginsenosides), herbs for detoxification (Goldthread Rhizome extract) and herbs activating blood circulation and detoxification (Rhubarb alcohol extract and Polygonum Cuspidatum extract). The results indicated the superior effect of Rhubarb alcohol extract and Polygonum Cuspidatum extract on stabilizing vulnerable plaque, showing the “class effect” of herbs activating blood circulation and detoxification in inhibiting inflammatory reaction and stabilizing plaque. 

The function of ITIH4 in European Molecular Biology Laboratory-The European Bioinformatics Institute (EMBL-EBI) showed that ITIH4 is a type II acute-phase protein (APP) involved in inflammatory responses to trauma. And it may also play a role in liver development and regeneration [50]. Inter-alpha-trypsin inhibitor (ITI) family proteins are all composed by light chain (bikunin) and at least 6 heavy chains. Lots of researches have proved that bikunin could inhibit the activity of protease, but little studies have paid attention to heavy chains of ITI. In 2000, Japanese scholar Choi-Miura et al. found that inter-alpha-trypsin inhibitor family heavy chain-related protein (IHRP) could inhibit the aggregation and phagocytosis of actins in polymorphonuclear leukocyte, implying that IHRP might be a new APP involved in inflammatory responses [51]. In 2004, Fujita et al. showed that genetic locus mutation of ITIH4 might be one of possible factors for dyslipidemia [52]. Then, Piñeiro et al. proved that ITIH4 was a new APP isolated from cattle during experimental infection [53]. Recently, Kashyap et al. found that ITIH4 showed high expression in normal subjects but no expression or little expression in patients with acute ischemic stroke (AIS), and this protein could return to normal level in blood serum gradually as the patients were getting better. The scholars considered that ITIH4 was a novel biomarker in inflammatory responses for AIS due to its close relationship with S-100 *β*-*β*, neuron specific enolase (NSE), interleukin-2 (IL-2), and interleukin-10 (IL-10) expression [54].

Results in this study showed that the group of activating blood circulation and detoxification could significantly increase ITIH4 expression and decrease FGA expression compared with the group of activating blood circulation and indicated that ITIH4 and FGA might be potential protein biomarkers for “toxin syndrome” of CHD. ITIH4 was further demonstrated in a nested case-control study, indicating its potential role as a new prewarning biomarker in stable CHD patients. 

Before recommending the conclusion of this study to clinical practice, we have to consider the following weaknesses. We cannot ascertain whether the tendency of these two polypeptides is always the same in the process of “toxin syndrome” development. Therefore, a large prospective cohort study collecting information in more time points is necessary.

## 6. Conclusion

ITIH4 might be a new potential biomarker of CHD “toxin syndrome” in TCM, indicating the potential role as a prewarning biomarker in stable CHD patients. 

## Figures and Tables

**Table 1:** Baseline information of two groups in RCT.

Groups	Group A	Group B
Age		
Minimum value (years)	48	42
Maximum value (years)	74	75
Mean value (years)	61.94 ± 8.41	61.24 ± 9.86
Sex		
Male (proportion)	22 (71%)	25 (86.2%)
Female (proportion)	9 (29%)	5 (13.8%)
Angina score	14.42 ± 4.86	14.89 ± 4.63
Primary symptom score of TCM	17.97 ± 6.74	18.94 ± 5.64
BSS score	10.89 ± 4.62	10.59 ± 3.38
Past history		
Hypertension (*N*)	18	16
Diabetes (*N*)	7	9
Dislipidemia (*N*)	11	12
Stroke (*N*)	2	4
Peripheral vascular atherosclerosis (*N*)	5	3
Old myocardial infarction (*N*)	4	1
Western medicine		
Aspirin (*N*)	31	30
Clopidogrel hydrogen sulfate (*N*)	31	30
Nitrates (*N*)	21	16
*β*-blocker (*N*)	28	30
ACEI/ARB (*N*)	19	17
CCB (*N*)	3	9
Low molecular weight heparin (*N*)	16	19
Statins (*N*)	30	29
UA risk stratification		
Low risk (*N*)	0	0
Mediate risk (*N*)	26	24
High risk (*N*)	5	6
Number of stenosed coronary vessel		
1 vessel (*N*)	8	12
2 vessels (*N*)	12	7
3 vessels (*N*)	11	11
Lesions nature		
De novo (*N*)	28	26
Restenosis (*N*)	3	4
Stent type		
Sirolimus-eluting stent (*N*)	21	21
paclitaxel-eluting stent (*N*)	8	6
Mixed drug-eluting stents (*N*)	2	3
Total length of stents	22.94 ± 7.23	21.67 ± 9.69

Note: group A patients have taken the Xiongshao capsule; group B patients have taken the Xiongshao capsule and Huanglian capsule.

**Table 2:** Comparison of before and after treatment in group A ($\overset{-}{X}\;\pm \;S$).

Mass (Da)	Ave ± StdDev (A-Q)	Ave ± StdDev (A-H)	*P*
1076.12	3.77 ± 2.35	2.54 ± 1.24	0.016099
1136.37	7.25 ± 3.12	5.42 ± 2.08	0.008065
1205.62	9 ± 3.32	6.92 ± 4.43	0.018784
1329.42	14.44 ± 7.31	10.26 ± 4.92	0.000268
1348.81	10.41 ± 4.08	7.09 ± 2.89	0.000806
1464.89	24.93 ± 13.64	13.52 ± 6.44	0.000293
1519.06	18.49 ± 8.17	11.39 ± 5.92	0.000111
1544.61	30.66 ± 17.33	19.41 ± 16.3	0.011662
1616.74	33.35 ± 22.17	18.7 ± 9.09	0.002143
2209.31	37.24 ± 18.84	25.45 ± 17.84	0.01935
2279.51	50.15 ± 17.26	40.99 ± 14.08	0.018232
2644.01	30.08 ± 20.39	22.42 ± 14.8	0.000347
2660.01	288.15 ± 231.67	206.03 ± 161.86	0.003587
2862.02	77.55 ± 78.74	45.15 ± 29.33	0.00951
3261.70	125.1 ± 63.45	159.89 ± 62.44	0.043161
3277.49	45.57 ± 23.64	59.7 ± 29.1	0.015557
4053.87	71.26 ± 38.16	95.63 ± 53.55	0.047052
4710.25	15.53 ± 5.5	12.84 ± 4.04	0.028146
4936.10	17.25 ± 15.28	9.77 ± 2.99	0.015955
4964.02	147.4 ± 184.34	59.95 ± 34.25	0.019728
5807.76	52.08 ± 23.63	70.92 ± 24.68	0.010841
5822.51	22.33 ± 13.95	31.77 ± 10.34	0.007368
5904.69	881.7 ± 598.83	1266.04 ± 415.59	0.00471
6049.22	27.36 ± 10.77	32.41 ± 10.1	0.023583

Note: paired sample *t* test was used, 2-tailed, and *P* < 0.05 was considered significant; Ave: peak area/intensity average; StdDev: standard deviation of the peak area/intensity average; A-Q: before treatment in group A; A-H: after treatment in group A.

**Table 3:** Comparison of before and after treatment in group B ($\overset{-}{X}\;\pm \;S$).

Mass (Da)	Ave ± StdDev (B_Q)	Ave ± StdDev (B_H)	*P*
1616.74	32.83 ± 18.26	23.95 ± 12.74	0.033415
2209.31	29.04 ± 14.35	21.23 ± 10.27	0.022373
2881.04	49.8 ± 18.98	38.19 ± 10.15	0.010917
3207.37	56.15 ± 17.06	47.84 ± 12.01	0.023899
4053.87	60.23 ± 22.82	83.04 ± 42.14	0.010323
4266.31	35.94 ± 19.99	45.73 ± 19.4	0.020676
4279.95	18.9 ± 8.79	27.23 ± 23.79	0.025866
4817.85	20.34 ± 17.41	11.15 ± 7.93	0.012491
4936.10	21.91 ± 27.22	10.29 ± 3.94	0.02628
5066.25	25.87 ± 7.61	32.1 ± 11.2	0.019568
5248.63	21.01 ± 5.89	25.23 ± 8.04	0.025751
6378.01	47.98 ± 47.92	28.92 ± 10.34	0.045958
7833.86	10.85 ± 2.21	12.97 ± 4.82	0.040051
9064.40	30.36 ± 8.86	35.83 ± 10.12	0.012614
9290.26	907.98 ± 442.14	1126.48 ± 455.76	0.023601

Note: paired sample *t*-test was used, 2-tailed, and *P* < 0.05 was considered significant; Ave: peak area/intensity average; StdDev: standard deviation of the peak area/intensity average; B-Q: before treatment in group B; B-H: after treatment in group B; overstriking mass: unique to group B.

**Table 4:** Comparison of difference between before and after treatment in the two groups.

Mass (Da)	Ave ± StdDev (A_H − A_Q)	Ave ± StdDev (B_H − B_Q)	*P*
1329.42	−4.18 ± 5.02	0.4 ± 6.86	0.005918
1519.06	−7.1 ± 7.9	−2.47 ± 9.08	0.046435
2660.01	−82.12 ± 130.27	3.85 ± 184.48	0.047102
2881.04	4.86 ± 16.64	−11.62 ± 23.4	0.003442
**3207.37**	**6.56 ± 21.34**	−**8.31 ± 19.09**	**0.008696**
3277.49	14.13 ± 27.75	−9.87 ± 33.72	0.005087
3972.15	−8.69 ± 22.59	3.47 ± 15.5	0.02561
**4279.95**	−**2.49 ± 14.27**	**8.32 ± 19.41**	**0.020196**
5066.25	−0.43 ± 10.97	6.23 ± 13.8	0.049446
5807.76	18.84 ± 34.89	−2.2 ± 36.2	0.031283
5822.51	9.44 ± 16.49	−7.75 ± 32.03	0.013558
6088.68	8 ± 28.11	−8.21 ± 25.58	0.029233

Note: paired sample *t*-test was used, 2-tailed, and *P* < 0.05 was considered significant; Ave: peak area/intensity average; StdDev: standard deviation of the peak area/intensity average; “A_H − A_Q”: subtraction between after and before treatment in group A; “B_H − B_Q”: subtraction between after and before treatment in group B; overstriking mass: unique to “Toxin.”

**Table 5:** Peptides identification unique to “Toxin”.

Mass	IPI	Gene_Symbol	Amino acid sequence
4280.13 Da	IPI00218192.3	ITIH4 Isoform 2 of inter-alpha-trypsin inhibitor heavy chain H4	R.NVHSAGAAGSRMNFRPGVLSSRQLGLPGPPDVPDHAAYHPF.R

3206.42 Da	IPI00021885.1	FGA Isoform 1 of Fibrinogen alpha chain precursor	K.SSSYSKQFTSSTSYNRGDSTFESKSYKM*.A

**Table 6:** ITIH4 expression between ACEs group and matched group.

Groups	Patients	MOD	*P*
ACEs group	10	8.41 ± 4.04	0.027
Matched group	10	11.57 ± 5.34	

Note: paired sample *t*-test was used, 2-tailed, and *P* < 0.05 was considered significant; MOD: mean optical density.
